# Cytarabine-based induction immunochemotherapy in the front-line treatment of older patients with mantle cell lymphoma

**DOI:** 10.1038/s41598-019-49776-9

**Published:** 2019-09-19

**Authors:** Sumita Ratnasingam, Joshua Casan, Jake Shortt, Eliza Hawkes, Michael Gilbertson, Zoe McQuilten, George Grigoriadis, Kay Thwe Htun, Swe Myo Htet, Philip Campbell, Khai Li Chai, Hang Quach, Sushrut Patil, Stephen Opat

**Affiliations:** 1 Department of Haematology, Monash Health, Melbourne, Australia; 2 Department of Medical Oncology and Clinical Haematology, Olivia Newton John Cancer and Wellness Centre, Austin Hospital, Melbourne, Australia; 3 Department of Medical Oncology, Eastern Health, Melbourne, Australia; 4 Department of Haematology, Alfred Health, Melbourne, Australia; 5 Department of Haematology, Andrew Love Cancer Centre, University Hospital Geelong, Geelong, Australia; 6 Department of Haematology, St Vincent’s Hospital, Melbourne, Australia; 7 School of Clinical Sciences, Faculty of Medicine, Nursing & Health Sciences, Monash University, Melbourne, Australia

**Keywords:** B-cell lymphoma, B-cell lymphoma

## Abstract

The role of cytarabine-based induction and autologous stem cell transplantation (ASCT) in front-line treatment of younger patients with mantle cell lymphoma (MCL) is well established, however the utility of intensive approaches in older patients remains unclear. This retrospective study compared first line treatment outcomes in patients aged 60 years or more, treated at six tertiary centres between 2000–2015. 70 patients included had a median age of 69 (60–91) and most (94%) demonstrated advanced stage disease. Treatment regimens included: R-CHOP-like (n = 39), alternating R-CHOP/R-DHAC (n = 10), R-HyperCVAD/R-MA (n = 7), R-CHOP/Cytarabine (Nordic Protocol) (n = 10) and other (n = 4). 16 patients underwent an ASCT. The median follow-up for surviving patients was 37 months. Compared to R-CHOP-like therapies, cytarabine-based regimens were associated with an improved overall response rate (ORR) of 70% vs 33% (p < 0.001) and overall survival (OS) (HR 0.541, [0.292–1.001], p = 0.05). No difference in efficacy between different cytarabine-based regimens was detected, but R-HyperCVAD/R-MA was associated with increased hospitalisation and transfusion requirements. Patients undergoing ASCT demonstrated an improved median OS (HR 0.108 [0.015–0.796], p = 0.029) but were significantly younger. These results reaffirm the use of cytarabine in MCL for selected patients aged over 60. Such regimens should be strongly considered for this population in frontline therapy.

## Introduction

Since mantle cell lymphoma (MCL) was first recognised as a specific entity in 1992, patient outcomes have continued to improve, with increasing evidence favouring an intensified management strategy that incorporates both cytarabine-based induction immunochemotherapy and consolidative autologous stem cell transplantation (ASCT)^1–3^. However, the evidence underpinning this strategy is principally derived from young and favourable-risk patient cohorts that do not reflect the real-world MCL population, in which the median age at diagnosis exceeds 73 years^4^. Accordingly, there is a paucity of evidence to guide management decisions for older and comorbid patients, in whom the roles of cytarabine and ASCT have not been established. In considering this evidence gap, we undertook a retrospective review of older (>60 years) MCL patients receiving upfront treatment across six Australian centres, and sought to investigate outcomes of intensive management compared to R-CHOP-like therapy.

## Methods

This retrospective study was approved as low risk by the Human Research Ethics Committee (HREC) of Monash Health. De-identified data were collected from hospital records, and individual patient consent was deemed unnecessary by the HREC. Each participating site received independent approval from their respective HREC (see Supplementary Information). All data collection was undertaken in accordance HREC guidelines and regulations.

Chronologically sequential cases of treatment-naïve MCL aged >60 years, treated at six participating institutions were identified from prospectively maintained institutional databases between 2000–2015.

Eligible patients had MCL diagnosed according to WHO 2008 criteria, confirmed by either fluorescence *in situ* hybridization (FISH) for t(11;14) translocation or immunohistochemistry for cyclin D1 expression. Only cases with adequate datasets; including baseline characteristics, treatment regimens and outcome, were included. Patients were excluded if they were treated with palliative intent due to frailty or had indolent disease managed with an observational approach.

Data were obtained from hospital medical records and laboratory and radiology information systems and included patient demographic characteristics; Eastern Cooperative Oncology Group (ECOG) performance status (PS); histological diagnosis; radiological findings; staging investigations, including bone marrow biopsy, serum lactate dehydrogenase (LDH); therapy received, and outcomes. Extensive toxicity data were not collected but surrogate markers of toxicity including admission length, ICU admission rate and blood product transfusion requirements were recorded.

The primary endpoint assessed was overall survival (OS) with secondary endpoints of overall response rate (ORR) and complete response (CR) rate, progression free survival (PFS) and toxicity. The variables examined for impact on survival were age, sex, ECOG PS, serum LDH, mantle cell lymphoma international prognostic index (MIPI) score and treatment received. Statistical analysis was performed using SPSS statistical software with 2-tailed Fisher’s exact test for contingency tables and Kaplan-Meier survival curves with comparison using log-rank (Mantel-Cox) test. Multivariate analysis was performed using Cox regression. For the comparison of chemotherapy regimens, patients undergoing ASCT were censored at the time of transplant.

All datasets analysed during the current study are available from the corresponding author on reasonable request.

## Results

Seventy patients met the eligibility criteria and were included in the final analysis. Baseline characteristics are summarised in Table 1. The median age was 69 years (60–91) and 41% of patients were over 70 years old. There was a strong male predominance (74%) and most patients demonstrated good performance status (ECOG 0–1; 90%). However, other adverse risk features were prevalent including advanced stage (94%), an intermediate (25%) or high MIPI score (69%) and extra-nodal involvement (80%). Patients were predominantly categorised by regimen as ‘R-CHOP-like’ (included R-CHOP n = 34, R-CVP n = 4, and R-CEOP n = 1) or ‘Cytarabine-containing’. The few remaining patients (n = 4) not receiving these treatments were classified as “Other” (Table 2).

**Table 1:** Baseline characteristics.

	ARA-C based	R-CHOP-like	Other	P value
**Sex**				
Male	19	30	3	0.578
Female	8	9	1	
**Age**				
<70 years	15	25	2	0.328
≥70 years	12	14	2	
**Stage**				
Limited	2	2	0	0.999
Advanced	25	37	4	
**LDH** > **ULN**				
Yes	6	14	1	0.508
No	18	21	3	
Unknown	3	4	0	
**ECOG** > **1**				
Yes	3	4	0	0.489
No	24	33	4	
Unknown	1	1	0	
**MIPI Score**				
Low	1	2	1	0.812
Intermediate	8	8	0	
High	16	26	3	
Unknown	2	2	0	
**ASCT**				
Yes	7	8	1	0.411
No	20	31	3	

LDH indicates lactate dehydrogenase; ULN indicates upper limit of normal.

ECOG indicates performance status as determined by the Eastern Cooperative Oncology Group.

MIPI indicates mantle cell lymphoma international prognostic index.

ASCT indicates autologous stem cell transplant.

Ara-C based regimens are cytarabine containing chemotherapies such as R-HyperCVAD/R-MA, R-CHOP/cytarabine, R-CHOP/R-DHAC.

**Table 2:** Types of Chemotherapy.

Type of Chemotherapy	Number of patients (n = 70)
R-CHOP/R-DHAC^ψ^	10
R-HyperCVAD/R-MA	7
R-CHOP/ Ara-C	10
R-CHOP	34
R-CHOP like*	5
Other	4

Types of chemotherapy: R-CHOP/R-DHAC (rituximab, cyclophosphamide, doxorubicin, vincristine, prednisolone; rituximab, dexamethasone, cytarabine, carboplatin); R-HyperCVAD/R-MA (rituximab, cyclophosphamide, doxorubicin, vincristine, dexamethasone, methotrexate, cytarabine); R-CHOP/Ara-C (rituximab, cyclophosphamide, doxorubicin, vincristine, prednisolone, cytarabine);

*R-CHOP like includes: R-CEOP (rituximab, cyclophosphamide, etoposide, vincristine, prednisolone); R-CVP (rituximab, cyclophosphamide, vincristine, prednisolone).

^Ψ^R-CHOP/R-DHAC^6^ is similar to R-CHOP/R-DHAP^5^ chemotherapy with the exception of carboplatin 300 mg/m^2^ being infused over an hour, rather than cisplatin 100 mg/m^2^ continuous infusion over 24 hours.

The median duration of follow-up of surviving patients was 37 months (range 7–129); at the time of analysis, 43 patients were alive, and 27 patients had died. The overall median PFS was 45 months (range 35–55) and median OS was 67 months (range 54–81).

There were no differences in sex, age, stage, LDH, performance status or MIPI score between patients who received cytarabine-based, CHOP-like or other chemotherapy (Table 1). However, those who received an ASCT were significantly younger (median age 64 vs 71, p = 0.01). 15 of 42 (36%) patients under 70 years of age underwent ASCT compared to only 1 of 28 (4%) above the age of 70.

Superior response was evident for cytarabine-based therapy compared to R-CHOP-like therapy with ORRs of 70% (19/27) vs 33% (13/39) (p < 0.001) and CR rates of 67% (18/27) vs 21% (8/39) (p < 0.001).

**Figure 1:**
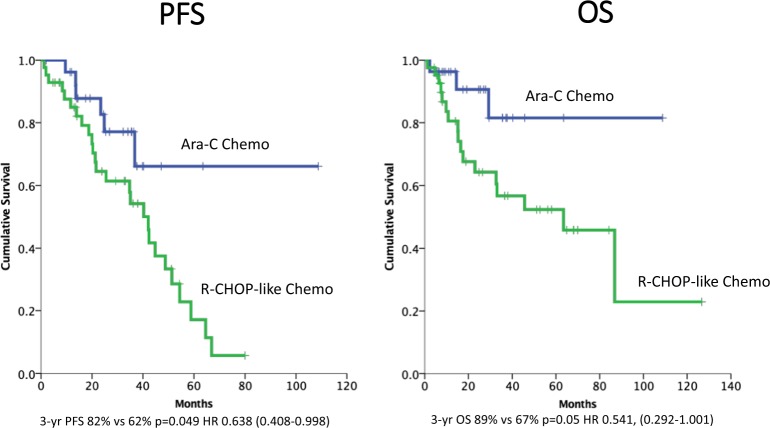
Progression free and overall survival by chemotherapy regimen on univariate analysis.

Patients receiving cytarabine demonstrated improved PFS (HR 0.638 [95% CI:0.408–0.998], p = 0.049) on multivariate analysis, with median PFS not reached for cytarabine recipients compared to 42 months for those treated with R-CHOP-like regimens (p = 0.049) (Fig. 1).

Treatment regimen was associated with OS in a univariate model (Fig. 1 and Table 3) with patients who received cytarabine demonstrating superior OS (3-year OS 89% *vs*. 67% HR 0.541, [0.292–1.001], p = 0.05). Age, sex, stage, LDH, and MIPI score did not influence OS. Patients with poor performance status (ECOG > 1) showed a trend toward inferior OS (3-year OS 38% *vs*. 76% HR 2.767 [0.936–8.182], p = 0.055). Although treatment group did not retain significance in a multivariate model (Table 4), there was a trend toward improved OS in cytarabine recipients (HR 0.347 [0.117–1.027], p = 0.056).

**Table 3:** Univariate Analysis.

	Progression Free Survival			Overall Survival		
	Hazard Ratio	95% confidence Interval	P	Hazard Ratio	95% confidence Interval	P
**Sex**						
Female (18) Male (52)	0.677	0.290–1.584	0.366	1.094	0.455–2.628	0.842
**Age**						
<70 years (42), ≥ 70 years (28)	1.338	0.633–2.826	0.445	0.660	0.300–1.452	0.305
**Stage**						
Limited (4) Advanced (66)	0.593	0.140–2.505	0.477	0.418	0.056–3.119	0.395
**Lactate dehydrogenase**						
Above normal (21) Below normal (42), Unknown (7)	0.709	0.331–1.519	0.376	0.670	0.292–1.536	0.344
**Performance status (ECOG)**						
0–1 (62), ≥2 (7), Unknown (1)	1.092	0.322–3.706	0.888	2.767	0.936–8.182	0.055
**MIPI Score**						
Low (4), Intermediate (16), High (45)	1.092	0.322–3.706	0.888	3.128	0.409–23.940	0.272
**Chemotherapy**						
R-CHOP-like (39), Ara-C containing (27), Other (4)	0.638	0.408–0.998	0.049	0.541	0.292–1.001	0.05

**Table 4:** Multivariate Analysis.

	Progression Free Survival			Overall Survival		
	Hazard Ratio	95% confidence Interval	p	Hazard Ratio	95% confidence Interval	p
**Performance status (ECOG)**						
0–1 (62), ≥2 (7), Unknown (1)	—	—	—	2.222	0.732–6.750	0.159
**Chemotherapy**						
R-CHOP-like (39), Ara-C containing (27), Other (4)	0.635	0.406–0.993	0.047	0.347	0.117–1.027	0.056

Patients receiving R-HyperCVAD/R-MA suffered greater toxicity necessitating longer hospitalisation and more transfusion support compared to other cytarabine-based therapy (Table 5): median admission length 54 days (31–72) *vs*. 19 days (2–77) (p < 0.001); median number of red cell units 16 (7–24) *vs*. 2 (0–16) (p < 0.001); median number of platelet pools 6 (2–15) *vs*. 0 (0–11) (Table 5) (p < 0.001). There were no significant differences for ICU admissions or platelet transfusion requirements between R-CHOP and non-R-HyperCVAD/R-MA cytarabine-containing regimens, but the latter were associated with higher median admission days (19 vs 0) and marginally higher red cell transfusion demand at a median of 2 units (0–15) vs 0 units (0–46) (p < 0.05). One patient (1.4%) receiving R-CHOP/R-DHAC died due to sepsis.

**Table 5:** Hospitalisation and Blood Product administration.

	R-CHOP/R-DHAC (n = 10)	R-HyperCVAD/R-MA (n = 7)	R-CHOP/Ara-C (n = 10)	R-CHOP like (n = 39)
Total days admission	20 (6–77)	54 (31–72)	15 (2–61)	0 (0–26)
ICU admissions	0	1 patient	1 patient	2 patients
Red cell units transfused	2 (0–5)	16 (7–24)	2 (0–16)	0 (0–46)
Pools of platelets transfused	0 (0–1)	6 (2–15)	0 (0–11)	0 (0–5)

## Discussion

The gold-standard treatment of MCL in elderly patients remains undefined, and as the median age at presentation exceeds 73 years, the optimal treatment for most MCL patients is unclear. Several landmark studies have established the utility of cytarabine-containing induction regimens, and the use of consolidative ASCT in young, fit patients. Phase II and III trials, performed by the Groupe d’Etude des Lymphomes de I’Adulte (GELA) consortium and the European MCL Network, deployed first-line combination R-CHOP (rituximab, cyclophosphamide, vincristine, doxorubicin, prednisolone) and R-DHAP (rituximab, dexamethasone, cytarabine, cisplatin) followed by ASCT, demonstrating impressive ORRs and OS with unequivocal superiority to R-CHOP alone^3,5^. The GELA study, in which 60 patients received 3 cycles of CHOP (rituximab added from the 3^rd^ cycle onwards), followed by 3 cycles of R-DHAP, demonstrated a CR rate of 96%, enabling 49 (81.7%) participants to proceed to ASCT. With a median follow up of 67 months, the 5-year OS was 75%^3^. The European MCL network phase III trial compared three cycles of induction R-CHOP alternating with three cycles of R-DHAP, to six cycles of R-CHOP, where all patients were planned for consolidative ASCT^5^. After a median follow up of 6.1 years, R-CHOP/R-DHAP was associated with median time to treatment failure (TTF) of 9.1 years *vs*. 3.9 years in the R-CHOP alone arm (Hazard ratio (HR) 0.56, p = 0.038) and improved progression free survival (PFS) (median not reached *vs*. 4.5 years; HR 0.45, p < 0.0001)^5^. However, participants in these studies were generally young (median age 56 in GELA study and 57 in European MCL network study), and median MIPI risk scores were low and intermediate respectively. Caution is therefore requisite when extrapolating conclusions from these studies for treating elderly, higher risk patients who were typical of our real-world cohort.

Randomised trials have investigated lower intensity immunochemotherapy in elderly MCL populations^6^. Bendamustine plus rituximab (BR) has demonstrated superior PFS over R-CHOP in two studies enrolling treatment-naïve elderly MCL patients^7,8^. At a median follow up of 45 months, MCL patients in the StiL (Study group indolent Lymphomas) trial (n = 94, median age 66 years) had a median PFS of 35.4 months with BR compared to 22.1 months with R-CHOP (HR 0.49, p < 0.0044)^7^. Similarly, the BRIGHT study compared BR to R-CHOP or R-CVP in treatment naïve indolent lymphoma, recruiting 74 MCL patients to the study^8^. BR was associated with improved CR rates (50% *vs*. 27%, p = 0.017) and 5-year PFS (39.7% *vs*. 14.2%, HR 0.40 [0.21–0.75], p = 0.0035) in MCL patients, when compared to R-CHOP or R-CVP^9^. Consequently, BR is now the considered by many as the default regimen of choice in elderly MCL. Importantly, at the time of data collection, bendamustine was not available as a subsidised treatment in Australia, and the absence of this comparator is a limitation of our study. However, given the proven potency of cytarabine in MCL, its utility in elderly patients should not go unexplored because of the availability of BR. Indeed, incorporation of cytarabine has been studied at a low dose (800 mg/m^2^) in a Phase II study, in which it was delivered in combination with bendamustine and rituximab (R-BAC)^10^. The median patient age was 70 and 50% of participants had relapsed or refractory disease. Though highly efficacious for treatment-naïve MCL (95% CR and 2-year PFS 95%), grade 3/4 haematological toxicity occurred in 87% of patients^10^. A subsequent study from the same group reduced the cytarabine dose further (500 mg/m^2^ on days 2–4) in elderly patients (median age 71). The dose reduction somewhat mitigated toxicity, though myelosuppression remained considerable (grade 3–4 haematological toxicity in 49% of patients), and safety was gained with some cost of efficacy (PFS rate of 76% at median follow up of 35 months)^11^. It is probable that the bendamustine back-bone in R-BAC constrains the deliverable dose of cytarabine, which is markedly lower than that in R-DHAP (4000 mg/m^2^ on day 2).

Given the significant association of younger age with ASCT in our study, we have not presented these data in further detail. However, the role of ASCT for MCL in elderly patients was reported by the European Bone Marrow Transplant registry, who compared outcomes of patients aged more than 65 years (n = 69) with those younger (n = 655)^12^. Non-relapse mortality, ORR, PFS and OS were similar across the age groups, with 5-year PFS 29% and OS 61% for the older cohort^12^. Though not a randomised comparison, these data support the use of ASCT in selected older patients.

The administration of high doses of cytarabine in elderly patients with other haematological malignancies, such as acute myeloid leukaemia, has proven challenging due to high rates of severe toxicities, and frequent simultaneous co-administration of additional myelosuppressive agents^2,13^. Accordingly, many clinicians are reluctant to adopt the use of cytarabine for routine care of elderly MCL patients; the minimal prospective data further compounding this reluctance. The Finnish Lymphoma Group, have published a multicentre Phase 2 study of untreated, elderly MCL patients (median age = 74), in which high dose (cumulative dose of 20 g/m^2^) cytarabine-rituximab was added as alternating induction therapy with R-CHOP (cycles 1–5), followed by three cycles of fludarabine, rituximab and cytarabine (cycles 6–8), and two additional CHOPs (cycles 9–10). Patients that exhibited a response then received maintenance rituximab for 2 years^14^. The ORR was high (95%), and 87% of patients achieved CR or unconfirmed CR. 4-year PFS was 70% and OS 72%. Treatment-related mortality was only 2%, but 28.3% of patients were not able to complete the therapeutic course, and 17% of patients who achieved response discontinued induction therapy prematurely due to intolerance. The total median duration of inpatient admission was 30 days, which is greater than that with R-CHOP/RDHAC (20 days) but less than with HyperCVAD (54 days) in our dataset^14^. Given that fludarabine is no longer a routinely recommended component of MCL therapy, these results are not easily generalisable. More recently, Klener *et al*. report prospective data for alternating R-CHOP/R-Cytarabine in transplant ineligible patients^15^. This observational study reported on 73 patients with a median age of 70 years, and this cohort closely resembled our own in regard to the frequency of advanced stage disease and intermediate/high risk MIPI scores. With 4-year estimated PFS and OS of 51.3% and 68.6% respectively, the efficacy appears comparable to our findings. 63 patients (86.3%) received 2 g/m^2^ of cytarabine and 10 patients (13.7%) received 1 g/m^2^. Toxicity was considerable with grade 3/4 haematological and non-haematological adverse events occurring in 48% of patients respectively, although no treatment-related mortality occurred and 91.8% of patients completed induction^15^. Although this study had no comparator arm, these data show that a cytarabine-containing regimen can be safely delivered for transplant-ineligible patients and achieve impressive efficacy and survival rates. Our study reaffirms this conclusion and recapitulates the benefits of cytarabine in MCL for elderly, high-risk patients.

We demonstrate that cytarabine-based therapy is deliverable, with acceptable toxicity and superior efficacy compared to R-CHOP. Approximately 70% of patients receiving cytarabine-based immunochemotherapy remained in CR1 (complete remission following first line therapy) over a median follow up of 37 months, in comparison to 33% of patients treated with R-CHOP-like chemotherapy. The role of cytarabine in younger patients is well established and no longer controversial given existing data. We contend that our study demonstrates a sound rationale for extending cytarabine to elderly patients. Regimens that include cytarabine in alternating sequence or combination with R-CHOP, akin to those used in the GELA and European MCL network studies, appear to balance safety and efficacy well. In our cohort, patients who received R-CHOP/cytarabine or alternating R-CHOP/R-DHAC appeared to exhibit less toxicity but similar efficacy in comparison to those receiving R-HyperCVAD/R-MA. In the absence of evidence of benefit in randomised phase 3 trials it would seem prudent to avoid more toxic regimens such R-HyperCVAD/R-MA that are associated with longer inpatient stays and higher blood product support in older patients.

Our data are clearly not without limitation however; safety data are incomplete, the study was retrospective, and bendamustine-rituximab was unable to be included as a comparator. However, prospectively recruiting elderly and highly comorbid patient populations for clinical trials is extremely challenging, and such patients are rarely represented in landmark trials. Our ‘real world’ study mitigates some of the recruitment bias intrinsic to clinical trials and provides meaningful information for clinicians faced with such clinical scenarios in routine practice.

An expanding array of novel therapeutics continues to emerge, with many demonstrating great promise in MCL^16–34^. Agents interfering with B cell receptor, Nuclear factor Kappa B(NF-kB), Mammalian target of rapamycin (mTOR) and Phosphatidylinositol-3-kinase (PI3K) signalling pathways, the epigenetic regulation of gene transcription and pro-apoptotic BH3-mimetics are the subject of active and intensive study. The success of these agents in the relapsed and refractory setting undoubtedly heralds their application to front line management. However, the cost may prove prohibitive in many settings, and accessibility will remain limited for the foreseeable future. The efficacy and toxicity of frontline novel agents in combination with standard immunochemotherapy are yet to be established as outcomes from clinical trials are eagerly awaited. Accordingly, the best use of such targeted therapies remains undefined. Therefore, immunochemotherapy cannot yet be consigned to obsolescence, and efforts to optimise its use should not be abandoned.

Our study confirms the safety and tolerability of frontline cytarabine-based regimens in selected older MCL patients, suggesting these options should be strongly considered by physicians contemplating therapeutic decisions in this population.

## Supplementary information

Participating sites
