# Supplementary material for: Cytarabine-based induction immunochemotherapy in the front-line treatment of older patients with mantle cell lymphoma
Source: Sci Rep. 2019 Sep 19;9:13544. doi: 10.1038/s41598-019-49776-9 (PMC6753133; doi:10.1038/s41598-019-49776-9)
Supplement: Supplementary file 1 — Participating sites [file 41598_2019_49776_MOESM1_ESM.pdf]

# Cytarabine-based induction immunochemotherapy in the front-line treatment of older patients with mantle cell lymphoma

***Sumita Ratnasingam[1,5], *Joshua Casan[1,7], Jake Shortt[1,7], Eliza Hawkes[2,3], Michael Gilbertson[1], Zoe McQuilten[1], George Grigoriadis[1,4,7], Kay Thwe Htun[4], Swe Myo Htet[5], Philip Campbell[5], Khai Li Chai[6], Hang Quach[6], Sushrut Patil[4] and Stephen Opat[1,7]**

[1]Department of Haematology, Monash Health, Melbourne, Australia, [2]Department of Medical Oncology and Clinical Haematology, Olivia Newton John Cancer and Wellness Centre, Austin Hospital, Melbourne, Australia, [3]Department of Medical Oncology, Eastern Health, Melbourne, Australia, [4]Department of Haematology, Alfred Health, Melbourne, Australia, [5]Department of Haematology, Andrew Love Cancer Centre, University Hospital Geelong, Geelong, Australia, [6]Department of Haematology, St Vincent's Hospital, Melbourne, Australia, [7]School of Clinical Sciences, Faculty of Medicine, Nursing & Health Sciences, Monash University, Melbourne, Australia

*Equal first authors

Participating Sites

Lead site: Monash Health, Department of Haematology, Melbourne, Australia

Other sites:

- Olivia Newton John Cancer and Wellness Centre, Department of Medical Oncology and Clinical Haematology, Austin Hospital, Melbourne, Australia
- Eastern Health, Department of Medical Oncology, Melbourne, Australia
- Alfred Health, Department of Haematology, Melbourne, Australia
- Andrew Love Cancer Centre, Department of Haematology, University Hospital Geelong, Geelong, Australia
- St Vincent’s Hospital, Department of Haematology, Melbourne, Australia

The lead Human Research Ethic Committee (HREC) was that of Monash Health. All participating sites obtained independent approval from their respective HRECs.
